# The dual pathways of emotional labor in psychiatric nursing: the adaptive and maladaptive emotional strategies

**DOI:** 10.3389/fpsyt.2026.1719188

**Published:** 2026-03-23

**Authors:** Guoyong Huang, Yawen Zheng, Guanghui Shen, Haihong Wang, Lu Wang

**Affiliations:** 1 Wenzhou Seventh People’s Hospital, Wenzhou, China; 2 Lishui Second People’s Hospital, Wenzhou Medical University, Lishui, China; 3 Wenzhou People’s Hospital, Wenzhou, China

**Keywords:** burnout, emotional labor, LPA, network bridge analysis, psychiatric nursing

## Abstract

**Background:**

Psychiatric nurses engage in high levels of emotional labor, which can significantly influence their burnout and job performance. While prior research has linked emotional labor to burnout, the nuanced interplay between different emotional regulation strategies remains underexplored. This study examines the distinct roles of surface acting (modifying outward expressions without changing internal feelings) and deep acting (adjusting internal emotions to align with external expectations) in psychiatric nursing, identifying their differential associations on burnout through network bridge analysis and latent profile analysis.

**Methods:**

A cross-sectional survey was conducted among 199 psychiatric nurses in a mental hospital in Wenzhou, China. Emotional labor was assessed using the Emotional Labor Scale, and burnout was measured with the Maslach Burnout Inventory-GS. Network bridge analysis was applied to identify key connections between emotional labor strategies and burnout dimensions. LPA was applied to reveal distinct emotional labor patterns.

**Results:**

Surface acting emerged as the primary bridge linking emotional labor to burnout, displaying strong associations with emotional exhaustion and depersonalization. LPA identified four emotional labor profiles: *moderate emotional labor* (63.32%), *low emotional labor* (5.03%), *high emotional labor* (23.62%), and *deep acting dominant* (8.04%). Nurses in the *deep acting dominant* profile exhibited significantly lower burnout levels compared to other groups (*p* < 0.001, Cohen’s *d* = 0.90–1.28), suggesting that deep acting mitigates burnout risks.

**Conclusion:**

These findings highlight the maladaptive effects of surface acting and the protective role of deep acting. Targeted interventions fostering deep acting may enhance psychiatric nurses’ well-being and resilience. Future research should explore longitudinal shifts in emotional labor strategies.

## 1 Introduction

The nursing profession serves as a cornerstone of modern healthcare systems, providing essential patient care, emotional support, and clinical expertise across diverse medical settings. As the largest segment of healthcare professionals globally, nurses play a vital role in ensuring the quality, efficiency, and accessibility of healthcare services (1). Within this expansive profession, psychiatric nursing stands out as a specialized field dedicated to addressing the complex needs of individuals with mental health conditions. Advances in medical care have increased demands on nurses, requiring not only technical competence but also advanced emotional skills (2). Psychiatric nurses, in particular, face unique challenges, including frequent exposure to emotionally intensive interactions, patient aggression, and unpredictable behaviors. These conditions necessitate exceptional psychological resilience and emotional regulation skills (3). Despite their critical role in mental health care, psychiatric nurses often encounter substantial professional hurdles, such as high burnout rates, societal stigma, and limited recognition compared to other nursing specialties (4). For example, burnout prevalence among psychiatric nurses ranges from 21% to 67%, significantly higher than that observed in general nursing populations, a disparity that has been linked the intense emotional labor inherent in their work (5).

Emotional labor is a crucial component of nursing practice, especially in psychiatric settings where sustained emotional engagement is essential for managing complex patient interactions. It is defined as the process by which professionals regulate their emotional expressions to meet organizational and professional expectations (6). Emotional labor includes two primary components: surface acting, which involves modified outward expressions without changing internal feelings, and deep acting, where internal emotions are adjusted to align with external behaviors (7). In psychiatric nursing, these components are employed to help build therapeutic alliances with patients who may exhibit emotional instability, cognitive impairments, or challenges in interpersonal relationships (8). The intensity and persistence of these emotional demands distinguish psychiatric nursing from other medical fields, as nurses must continually manage their emotional responses to sustain trust and ensure high-quality care. However, prolonged engagement in emotional labor has been associated with negative outcomes, such as emotional exhaustion (9) and reducing well-being (10), and has been linked to higher level of burnout and job dissatisfaction (11). Understanding the mechanisms of emotional labor is essential for improving the occupational health of psychiatric nurses and optimizing their contributions to mental healthcare delivery.

Burnout is a significant occupational challenge in nursing, particularly pronounced in psychiatric settings due to heightened emotional demands, patient aggression, and chronic workplace stressors (12). It is typically characterized by three core dimensions: emotional exhaustion (a sense of being emotionally drained and overwhelmed by work), depersonalization (developing cynical or detached attitudes toward patients), and reduced personal accomplishment (experiencing diminished sense of professional efficacy and satisfaction) (13). Psychiatric nurses are at elevated risk of burnout compared to general medical nurses, owing to their routine exposure to emotionally intense interactions and the persistent stigma surrounding mental healthcare (14). Empirical studies have consistently demonstrated a strong association between high levels of emotional labor and burnout in nursing populations (15). Surface acting, which involves suppressing genuine emotions while displaying organizationally expected affect, is particularly taxing and often associated with emotional dissonance (16, 17). In contrast, deep acting, where individual actively modify their internal emotional states to match professional expectations, has been linked to lower burnout level and greater job satisfaction (18). Despite these findings, the longitudinal effects of emotional labor on burnout within psychiatric nursing remain inadequately explored. The Emotional Dissonance Theory (19) posits that psychological stress arises when there is a disconnect between internally felt emotions and outwardly expressed affect. This framework suggests that surface acting may be associated with elevated emotional dissonance by requiring continuous suppression of authentic emotions, thereby exacerbating emotional exhaustion and depersonalization, two key components of burnout (20, 21). Conversely, deep acting is theorized to promote alignment between internal emotions and external expressions, reducing emotional dissonance and mitigating burnout risk.

Although the relationship between emotional labor and burnout is well-documented, conventional analytical methods, such as regression-based models, often fail to capture the nuanced, multidimensional nature of this relationship. Emotional labor encompasses distinct components, each demonstrating distinct associations with burnout. This complexity necessitates the application of advanced methodologies to uncover the underlying dynamics and their implications for psychiatric nurses’ well-being. Grounded in Emotional Dissonance Theory, the present study adopts an innovative analytical approach, including network bridge analysis and latent profile analysis (LPA), to provide a comprehensive examination of these relationships. Specifically, this study aims to: (1) map the network structure linking emotional labor strategies (surface acting, deep acting, and natural expression) with burnout dimensions (emotional exhaustion, depersonalization, and reduced personal accomplishment); (2) identify latent profiles of emotional labor strategy use among psychiatric nurses; and (3) examine how these profiles are associated with burnout outcomes. By addressing these objectives, this research seeks to advance understanding of emotional labor’s role in psychiatric nursing and support the development of targeted interventions to mitigate burnout.

## 2 Methods

### 2.1 Participants

This study employed a cross-sectional design, with sample size estimation based on Kendall’s classic strategy (22), which recommends a sample size of 5–10 times the number of variables. The demographic questionnaire included 4 variables, the Emotional Labor Scale contained 14 variables, and the Maslach Burnout Inventory-General Survey (MBI-GS) included 15 variables, leading to a minimum required sample size of 165. Considering potential missing data or invalid responses, an additional 10% invalidity rate was assumed, resulting in a target sample size of 183.

Participants were recruited from a mental hospital in Wenzhou City in June 2024. The inclusion criteria were: (1) licensed nursing personnel; (2) adequate cognitive function and Chinese literacy; and (3) provision of written informed consent. The exclusion criteria included individuals who (1) were on extended leave or had recently resigned, (2) had a history of severe mental illness affecting cognitive function, (3) were unwilling to complete the survey or withdrew from the study, or (4) exhibited response patterns indicative of irregular answering behavior. A total of 245 participants were initially enrolled. After excluding responses with irregular answering patterns and those with a completion time of less than 120 seconds, 199 valid questionnaires remained, resulting in an effective response rate of 81.22%.

### 2.2 Procedures

This study used an electronic questionnaire platform for data collection. QR code posters linking to the survey were generated and distributed. The head nurse of each ward was responsible for evaluating and identifying individuals who met the inclusion criteria. Prior to the start of data collection, designated nursing personnel in each ward were gathered and provided with training to ensure that all measurement items were correctly understood. This training aimed to minimize response bias and improve the accuracy of data collection. Participants who met the eligibility criteria were invited to scan the QR code and complete the questionnaire in a quiet environment. To ensure data quality, responses with irregular answering patterns and completion times of less than 120 seconds were excluded from the final analysis.

### 2.3 Measurements

#### 2.3.1 Demographic information

Demographic information was collected using a self-developed questionnaire, which included information about participants’ gender (male/female), age (in years), education level (high school and below/junior college/bachelor’s degree), and years of work experience (in years).

#### 2.3.2 Emotional labor

Emotional labor was assessed by the Emotional Labor Scale developed by Yang et al. (2019) (23). This scale evaluates three key performance strategies of emotional labor: surface acting, deep acting, and natural expression. The scale consists of 14 items, with each dimension measured separately. Participants rated their responses using a 5-point Likert scale, ranging from 1 (strongly disagree) to 5 (strongly agree). Higher scores indicate a greater tendency to engage in the corresponding emotional labor strategy. Cronbach’s α in the present study were 0.91 for surface acting, 0.72 for deep acting, and 0.86 for natural expression.

#### 2.3.3 Burnout

Burnout was assessed by the Chinese version of the burnout scale revised by Chaoping et al (24). The scale measures three dimensions of burnout: emotional exhaustion, depersonalization, and low achievement. The scale consists of 15 items, rated on a 7-point Likert scale ranging from 0 (never) to 6 (every day), with higher scores on emotional exhaustion, cynicism and low achievement, indicating a greater degree of burnout. Cronbach’s α coefficients in the current study were 0.96 for emotional exhaustion, 0.94 for depersonalization, and 0.94 for low achievement.

### 2.4 Statistical analysis

All statistical analyses were performed using SPSS 26.0 (IBM Corp., Armonk, NY, USA) and R 4.2.2 (R Core Team). Descriptive statistics (means, standard deviations, frequencies, and percentages) were used to characterize the demographic variables, emotional labor, and burnout. Attrition analyses were conducted using chi-square tests for categorical variables and independent t-tests for continuous variables to examine potential differences between included and excluded participants. First, Pearson correlation coefficients were computed to examine bivariate relationships among demographic variables, emotional labor dimensions, and burnout components.

Second, Network analysis was conducted to visualize and quantify the connections between the emotional labor subdimensions and the burnout dimensions. Partial correlation matrices were constructed using the R packages “qgraph” and “networktools” (25). Bridge centrality measures (bridge strength, bridge betweenness, and bridge closeness) were computed to identify which nodes served as connectors between emotional labor and burnout. Bootstrap procedures were used to assess the stability of network edges and centrality metrics.

Third, latent profile analysis (LPA) was performed using the R packages “mclust”. Fourteen items from the Emotional Labor Scale were treated as continuous indicators to identify distinct profiles of emotional labor among the nurses. Competing solutions ranging from one to six profiles were compared, and model fit was evaluated through the Akaike information criterion (AIC), Bayesian information criterion (BIC), sample-size–adjusted BIC, entropy, and bootstrap likelihood ratio test (BLRT) (26). The solution with the lowest AIC and BIC, the highest entropy, and a significant BLRT was selected as the final model.

Finally, one-way analysis of variance (ANOVA) with Bonferroni-correction (27) *post-hoc* comparisons was conducted to examine burnout differences across the LPA profiles. Effect sizes were expressed as Cohen’s *d* and interpreted following standard guidelines (0.2 = small, 0.5 = medium, 0.8 = large) (28). All statistical tests were two-tailed, with *p* < 0.05 indicating statistical significance.

## 3 Results

### 3.1 Attrition analyses

Attrition analyses were conducted to examine potential differences in demographic characteristics between the included (*n* = 199) and excluded samples (*n* = 46). The results indicated no significant differences between the two groups in terms of gender (*χ*² = 1.40, *p* = 0.24), educational level (*χ*² = 1.83, *p* = 0.40), age (*t* = 1.05, *p* = 0.30), or work years (*t* = 1.12, *p* = 0.27).

### 3.2 Descriptive statistics

The study included 199 mental health nurses, with a mean age of 30.64 ± 6.13 years and average work experience of 8.62 ± 6.45 years. The majority of participants were female (93.47%, *n* = 186), and most held a bachelor’s degree (81.91%, *n* = 163). Other details are given in **Table 1**.

**Table 1:** Demographic characteristics of the participants (n = 199).

Variables	M ± SD	n (%)
Age	30.64 ± 6.13	
Gender		
Male		13(6.53)
Female		186(93.47)
Years of work experience	8.62 ± 6.45	
Educational level		
High school and below		3(1.51)
Junior college		33(16.58)
Bachelor’s degree		163(81.91)
Emotional labor		
Surface acting	20.57 ± 5.22	
Deep acting	13.54 ± 2.54	
Natural expression	10.06 ± 1.64	
Burnout		
Emotional exhaustion	15.52 ± 5.94	
Depersonalization	10.66 ± 4.95	
Low achievement	20.17 ± 7.94	

Correlation analysis revealed significant negative associations between years of work experience and emotional labor strategies, specifically with surface acting (*r* = -0.27, *p* < 0.001), deep acting (*r* = -0.26, *p* < 0.001), and natural expression (*r* = -0.15, *p* < 0.05). There were no significant associations between years of work experience and burnout. Regarding the relationships between emotional labor and burnout dimensions, surface acting showed significant positive correlations with emotional exhaustion (*r* = 0.44, *p* < 0.001) and depersonalization (*r* = 0.42, *p* < 0.001). Deep acting was negatively associated with low achievement (*r* = -0.23, *p* < 0.001), while natural expression also demonstrated a negative correlation with low achievement (*r* = -0.28, *p* < 0.001) (**Table 2**).

**Table 2:** Correlations matrix for the study variables.

Variables	1.	2.	3.	4.	5.	6.	7.	8.	9.	10.
1.Gender	1									
2.Educational level	0.07	1								
3.Age	0.10	0.18^**^	1							
4.Years of work experience	0.10	0.09	0.93^***^	1						
5.Emotional exhaustion	-0.15^*^	0.19^**^	-0.05	-0.09	1					
6.Depersonalization	-0.19^**^	0.10	-0.06	-0.11	0.85^***^	1				
7.Low achievement	0.02	0.03	-0.04	-0.09	0.09	0.16^*^	1			
8.Surface acting	-0.06	0.12	-0.22^**^	-0.27^***^	0.44^***^	0.42^***^	0.05	1		
9.Deep acting	-0.02	0.10	-0.21^**^	-0.26^***^	0.10	0.01	-0.23^***^	0.51^***^	1	
10.Natural expression	-0.02	0.02	-0.14^*^	-0.15^*^	-0.02	-0.06	-0.28^***^	0.34^***^	0.58^***^	1

^*^: *P* < 0.05; ^**^: *P* < 0.01; ^***^: *P* < 0.001.

### 3.3 Bridge network

**Figure 1:**
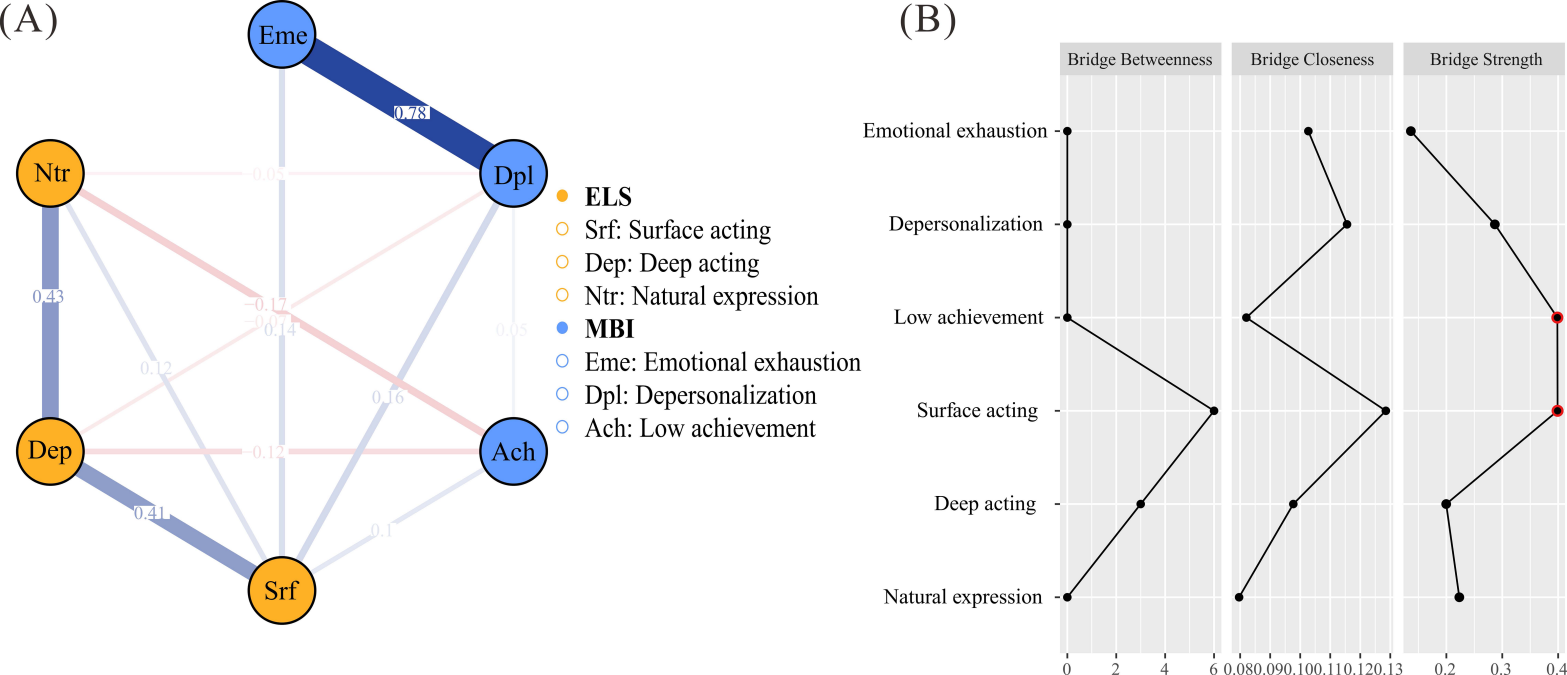
Bridge analysis of the network structure between emotional labor and burnout. **(A)** Network structure illustrating the relationships between emotional labor (surface acting, deep acting, and natural expression) and burnout (emotional exhaustion, depersonalization, and low achievement). Edge thickness represents the strength of the connections, with blue indicating positive associations and red indicating negative associations. **(B)** Bridge centrality displaying bridge betweenness, bridge closeness, and bridge strength. Surface acting and low achievement exhibit the highest bridge strength.

The network analysis examined the connections between the subdimensions of emotional labor (surface acting, deep acting, natural expression) and burnout (emotional exhaustion, depersonalization, low achievement). The constructed network included 6 nodes and 12 non-zero edges out of a possible 15, with a mean edge weight of 0.12. The bridge analysis, as illustrated in **Figure 1A**, surface acting (Srf) exhibited a positive connection with both emotional exhaustion (Emc) (weight edge = 0.14) and depersonalization (Dpl) (weight edge = 0.16). Additionally, surface acting demonstrated a positive connection with low achievement (Ach) (weight edge = 0.10). Conversely, Deep acting (Dep) negatively associated with Dpl (weight edge = -0.07) and negative connection with Ach (weight edge = -0.12). **Figure 1B** presents the bridge centrality analysis, highlighting the relative importance of different nodes in connecting emotional labor and burnout. Srf (bridge strength = 0.40) and Ach (bridge strength = 0.39) emerged as the primary bridge node, exhibiting the highest bridge strength.

### 3.4 Latent profile analysis of emotional labor

**Figure 2:**
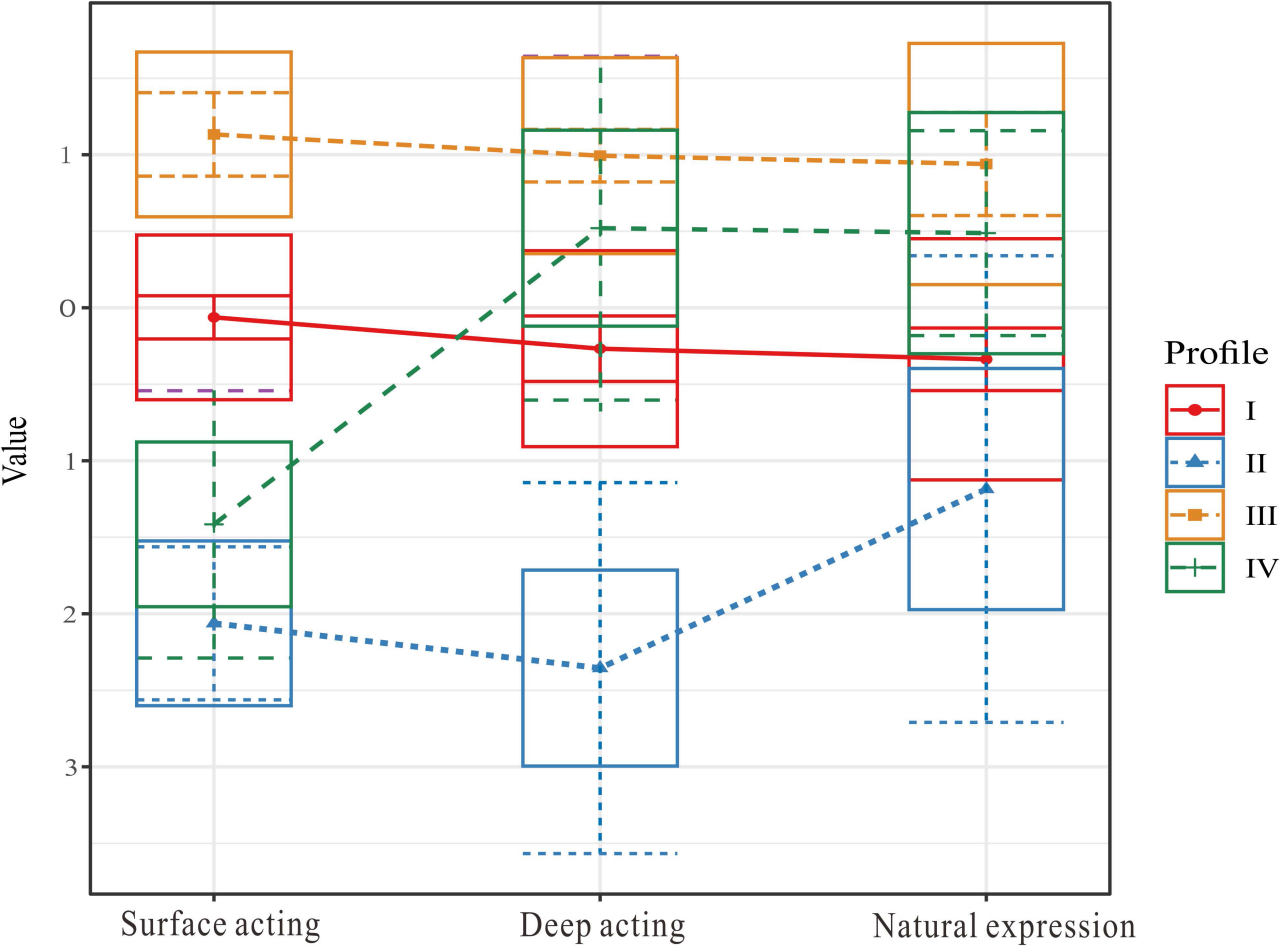
Four latent profiles of nurses’ emotional labor.

With 14 items of the nurses’ emotional labor scale as explicit indicators, 1 to 6 latent profile models were constructed, and the results are shown in **Table 3** and **Figure 2**. As the number of latent profiles increased from 1 to 6, the fitting indices AIC and BIC gradually decreased. Entropy decreases and then increases, reaching its high point in the four-profile model (entropy = 0.87). Moreover, BLRT were statistically significant in Classes 2, Classes 3, and Classes 4 while the p value of the BLRT was > 0.05 in the five-profile model. Therefore, the four-profile model was finally chosen as the best classification scheme in this study.

**Table 3:** Latent profiling of nurses’ emotional labor fitting indices.

Classes	AIC	BIC	Entropy	n_min	n_max	BLRT
1	1703.21	1722.96	1.00	1.00	1.00	
2	1645.26	1678.19	0.94	0.05	0.95	0.01
3	1560.26	1606.36	0.85	0.04	0.70	0.01
4	1537.76	1597.04	0.87	0.05	0.63	0.01
5	1542.58	1615.03	0.68	0.05	0.38	0.58
6	1519.40	1605.03	0.84	0.02	0.45	0.01

The four-profile model identified distinct emotional labor patterns among nurses, as detailed in **Table 4**. Profile I, labeled as “moderate emotional labor” represented the largest group (63.32%, *n* = 126), characterized by moderate levels of surface acting, deep acting, and natural expression. Profile II, identified as “low emotional labor” was the smallest group (5.03%, n = 10), showing consistently low scores across all dimensions. Profile III, termed “High Emotional Labor” comprised 23.62% (*n* = 47) of the sample and exhibited the highest scores across all dimensions. Notably, Profile IV, characterized as “Deep acting dominant” (8.04%, *n* = 16), displayed a distinctive pattern that differentiated it from other profiles: while maintaining relatively low surface acting, it showed markedly higher levels of deep acting and natural expression. This unique combination showed a more adaptive emotional labor pattern, where nurses rely more on internally modifying their feelings (deep acting) rather than changing their external expressions (surface acting).

**Table 4:** Four-profile of emotional labor patterns in nurses (N = 199).

Profiles	Simple size	Surface acting	Deep acting	Natural expression
Moderate emotional labor (Profile I)	126(63.32%)	20.20 ± 2.43	12.88 ± 1.53	9.52 ± 1.24
Low emotional labor (Profile II)	10(5.03%)	10.00 ± 2.87	7.40 ± 2.22	8.10 ± 2.03
High emotional labor (Profile III)	47(23.62%)	26.68 ± 2.97	16.15 ± 1.30	11.62 ± 1.38
Deep acting dominant (Profile IV)	16(8.04%)	12.19 ± 3.29	14.94 ± 2.35	10.88 ± 1.09

**Figure 3:**
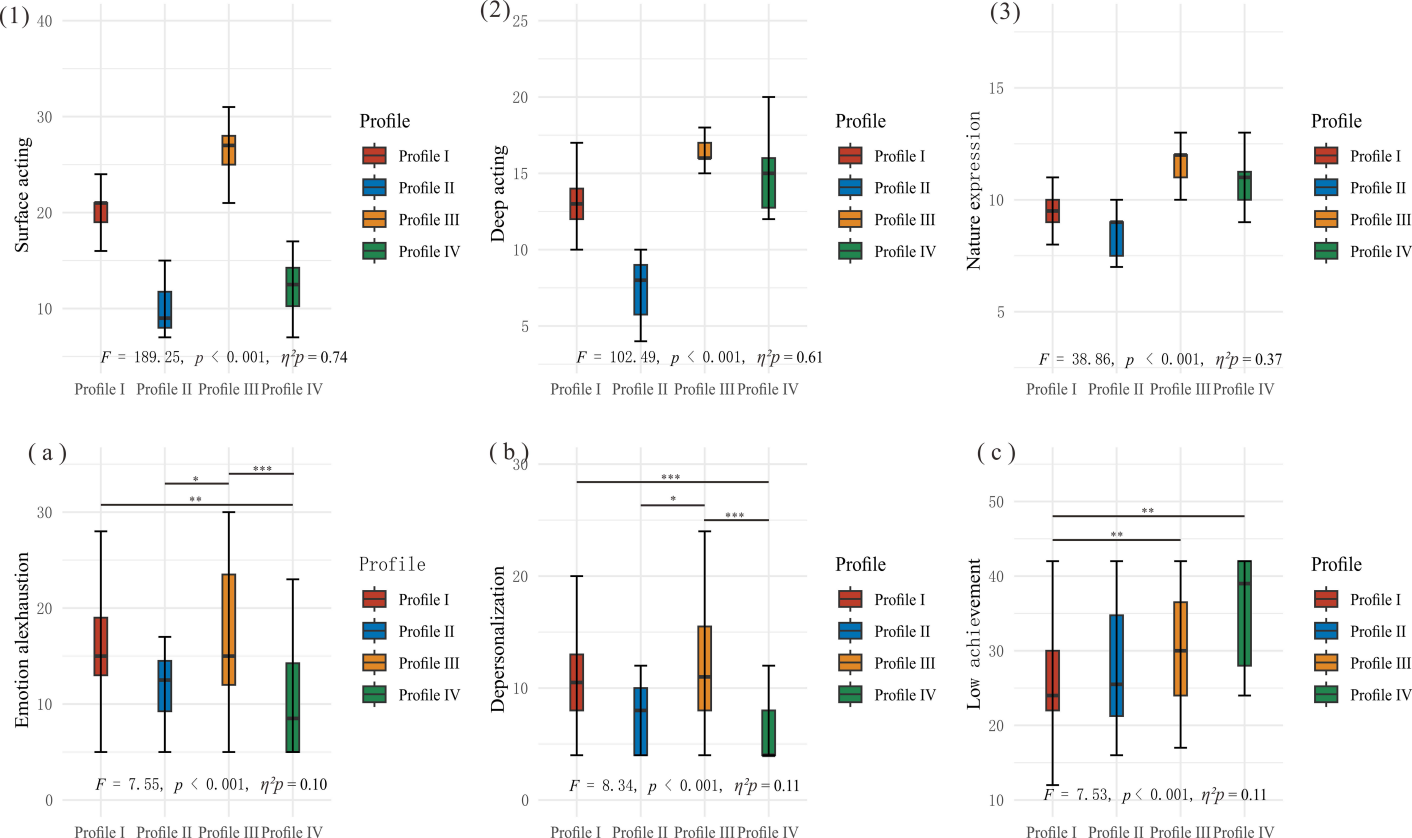
Differences in emotional labor and burnout dimensions across latent profiles. (1–3) Boxplots depicting differences in surface acting, deep acting, and natural expression across the four latent profiles of emotional labor; **(a–c)** Boxplots showing variations in emotional exhaustion, depersonalization, and low achievement among the profiles. Bonferroni correction for 6 tests was applied to adjust for multiple comparisons. *:P < 0.05; **:P < 0.01; ***:P < 0.001.

One-way ANOVA revealed significant differences among the four profiles in all three burnout dimensions: emotional exhaustion (*F* = 7.55, *p* < 0.001), depersonalization (*F* = 8.34, *p* < 0.001), and low achievement (*F* = 7.53, *p* < 0.001) (**Table 5**, **Figure 3**). *Post-hoc* comparisons with Bonferroni correction revealed distinctive patterns across profiles. The results are presented in **Table 5**. Deep acting dominant pattern exhibited significantly lower scores of emotional exhaustions compared to both moderate emotional labor pattern (*p* < 0.01, Cohen’s *d* = 0.90) and high emotional labor pattern (*p* < 0.001, Cohen’s *d* = 1.21), with large effect sizes. Similarly, in terms of depersonalization, deep acting dominant pattern showed significantly lower scores than both moderate emotional labor pattern (*p* < 0.001, Cohen’s *d* = 1.09) and high emotional labor pattern (*p* < 0.001, Cohen’s *d* = 1.28). The pattern continued in the low achievement dimension, deep acting dominant pattern demonstrated significantly lower levels compared to Profile I (*p* < 0.001, Cohen’s *d* = 0.95). In addition, high emotional labor pattern demonstrated significantly lower scores than moderate emotional labor pattern (*p* < 0.01, Cohen’s *d* = 0.63), indicated high deep acting engagement contribute to a greater sense of professional achievement.

**Table 5:** Analysis of variance in the burnout dimension of different emotional labor patterns.

Profiles	Profile I	Profile II	Profile III	Profile IV	*F*	*p*	Pairwise comparison (Cohen’s *d*)
Emotional exhaustion	15.74 ± 5.06	11.50 ± 4.22	17.45 ± 7.19	10.63 ± 5.85	7.55	< 0.001	IV< I^**^ (d =0.90) IV< III^***^(d=1.21) II< III^*^(d= 1.05)
Depersonalization	11.03 ± 4.667	7.50 ± 3.24	11.94 ± 5.42	5.94 ± 2.84	8.34	< 0.001	II< III^*^(d= 0.95) IV< I^***^(d=1.09) IV< III^***^(d=1.28)
Low achievement	21.96 ± 6.97	20.40 ± 9.65	17.17 ± 7.64	14.75 ± 10.31	7.53	< 0.001	III <I^**^(d= 0.63) IV< I^***^(d=0.95)

Pairwise Comparison: *Bonferroni correction for 6 tests.* ^*^: *P* < 0.05; ^**^: *P* < 0.01; ^***^: *P* < 0.001. Profile I: Moderate emotional labor; Profile II: Low emotional labor; Profile III: High emotional labor; Profile IV: Deep acting dominant.

## 4 Discussion

The present study investigated the complex relationship between emotional labor and burnout among psychiatric nurses, utilizing network bridge analysis and latent profile analysis to capture the multidimensional nature of these constructs. The findings provide novel insights into how distinct patterns of emotional labor are associated with burnout dimensions, thereby corroborating and extending prior research grounded in Emotional Dissonance Theory. By identifying four distinct emotional labor profiles and their differential relationships with burnout, the study underscores the pivotal role of emotional labor in psychiatric nursing and highlights adaptive strategies that may help alleviate occupational strain.

### 4.1 Network structure of emotional labor and burnout

The network analysis identified surface acting as a pivotal bridge node linking emotional labor to burnout, with strong positive connections to emotional exhaustion and depersonalization. This finding aligns with previous research showing that surface acting, characterized by the suppression of authentic emotions, amplifies emotional dissonance and depletes psychological resource (29). It is also aligned with Emotional Dissonance Theory, which posits that psychological stress arises from the discrepancy exists between experienced emotions and outwardly expressed affect. Persistent emotional dissonance resulting from surface acting appears to progressively exacerbate emotional exhaustion and depersonalization, two core components of burnout syndrome (30). The high bridge strength of surface acting underscores its role as a maladaptive strategy, consistent with the notion that prolonged inauthenticity in emotional expression heightens stress and detachment in psychiatric settings. Conversely, deep acting exhibited negative associations with depersonalization and low achievement, suggesting that modifying internal emotional states to align with professional demands may foster resilience and a sense of efficacy. These differential effects support Huppertz’s (2020) assertion that deep acting, by reducing emotional dissonance, serves as a protective mechanism against burnout—a finding particularly salient in the emotionally intense context of psychiatric care (31). In addition, the identification of surface acting and Low achievement as the primary bridge nodes highlights their central role in connecting emotional labor with burnout. This finding suggests that interventions targeting surface acting may have cascading benefits for reducing burnout across multiple dimensions, making it a strategic point for intervention.

### 4.2 Emotional labor profiles and their relationship to burnout

The LPA further enriched this understanding by delineating four emotional labor profiles among psychiatric nurses: “moderate emotional labor” (63.32%), “low emotional labor” (5.03%), “high emotional labor” (23.62%), and “deep acting dominant” (8.04%). Each profile exhibited unique patterns of burnout outcomes, providing insights beyond what traditional variable-centered approaches might reveal. The “deep acting dominant” profile (Profile IV), characterized by low surface acting but high deep acting and natural expression, demonstrated significantly lower levels of emotional exhaustion, depersonalization, and reduced personal accomplishment compared to other profiles. This finding supports the theoretical proposition that adopting deep acting as a primary emotional labor strategy promotes better occupational well-being (32, 33). The predominance of the “moderate emotional labor” (Profile I) suggests that most nurses adopt a balanced approach to emotional regulation, potentially reflecting an adaptive response to the chronic demands of their role. However, the high emotional labor profile, marked by elevated scores across all strategies, was associated with significantly greater emotional exhaustion and depersonalization compared to the deep acting dominant profile. This disparity reinforces the hypothesis that excessive reliance on surface acting, even when paired with deep acting, amplifies burnout risk due to sustained emotional dissonance (34). The “low emotional labor” profile (Profile II), while representing only a small portion of the sample (5.03%), showed moderate levels of burnout. This finding raises questions about potential disengagement or emotional withdrawal as a coping mechanism, which may protect against immediate emotional exhaustion but potentially compromise care quality and professional satisfaction over time (35).

## 5 Limitations

Several limitations of the present study should be acknowledged. First, the cross-sectional design precludes causal inferences regarding the relationship between emotional labor and burnout. Longitudinal research is needed to establish temporal precedence and elucidate how emotional labor profiles may shift over time in response to organizational factors, experience, and personal development. Second, the sample was drawn from mental hospitals in Wenzhou City, potentially limiting the generalizability of findings to psychiatric nurses in other regions. Emotional expression norms and professional role expectations are shaped by sociocultural contexts. In collectivistic cultural settings, where interpersonal harmony and emotional restraint are emphasized, nurses may experience distinct pressures to regulate emotional expression. These contextual factors may influence both the prevalence of specific emotional labor strategies and their associations with burnout. Therefore, caution is warranted when generalizing the present findings to healthcare systems operating under different cultural frameworks. The predominantly female sample also limits our understanding of potential gender differences in emotional labor patterns and burnout experiences among psychiatric nurses. Third, the relatively small size of some profiles necessitates caution in interpreting differences among these profiles. Replication with larger samples would enhance confidence in the stability and generalizability of these profiles. Finally, while this study focused on the internal dynamics of emotional labor strategies, it did not account for external moderators such as organizational support, objective workload, or individual coping resources. Future studies should adopt a more holistic framework by incorporating these variables to better understand the boundary conditions of adaptive emotional labor.

## 6 Conclusion

This study provides a comprehensive examination of the interplay between emotional labor and burnout among psychiatric nurses, leveraging network bridge analysis and LPA to address gaps in understanding the multidimensional nature of these constructs. The findings affirm that surface acting emerging as a critical driver of emotional exhaustion and depersonalization, while deep acting offers a protective buffer against these outcomes. The network analysis pinpointed surface acting as a key bridge node linking emotional labor to burnout, underscoring its maladaptive role in psychiatric settings where emotional demands are uniquely intense. The LPA identified four distinct emotional labor profiles. These results extend Emotional Dissonance Theory by demonstrating how emotional regulation strategies shape burnout trajectories, with implications for both theory and practice. The prominence of the moderate emotional labor profile suggests a baseline resilience among psychiatric nurses, yet the vulnerability of the high emotional labor group signals an urgent need for intervention. Conversely, the deep acting dominant profile’s lower burnout levels point to the potential of fostering internal emotional alignment as a sustainable approach to well-being.

## 7 Relevance for clinical practice

This study offers evidence-based recommendations for developing targeted interventions to address emotional labor among psychiatric nurses. First, training programs targeting the reduction of surface acting and promotion of deep acting strategies could be beneficial for reducing burnout risk. Such programs might include mindfulness-based interventions, which have shown promise in enhancing emotional awareness and regulation (36). Second, the existence of distinct emotional labor profiles suggests that interventions may be most effective when tailored to individual patterns of emotional labor rather than applying one-size-fits-all approaches. Third, the “deep acting dominant” profile could serve as an adaptive model for psychiatric nursing practice, with experienced nurses who exhibit this profile potentially serving as mentors for newer staff. Moreover, organizational policies that acknowledge and value the emotional aspects of psychiatric nursing work, rather than focusing exclusively on technical skills, may foster environments where genuine emotional engagement is supported and rewarded. This might include adjustments to workload distribution, provision of regular debriefing sessions, and creation of supportive team climates that normalize emotional challenges and collective problem-solving.

## Data Availability

The raw data supporting the conclusions of this article will be made available by the authors, without undue reservation.
